# Supplementary material for: The Construction and Exploration of a Comprehensive MicroRNA Centered Regulatory Network in Foxtail Millet (Setaria italica L.)
Source: Front Plant Sci. 2022 May 6;13:848474. doi: 10.3389/fpls.2022.848474 (PMC9121102; doi:10.3389/fpls.2022.848474)

T=Seita.3G274300.1_Q=Sit−miR171g_S=552

category=0_p=0.0200424732434205

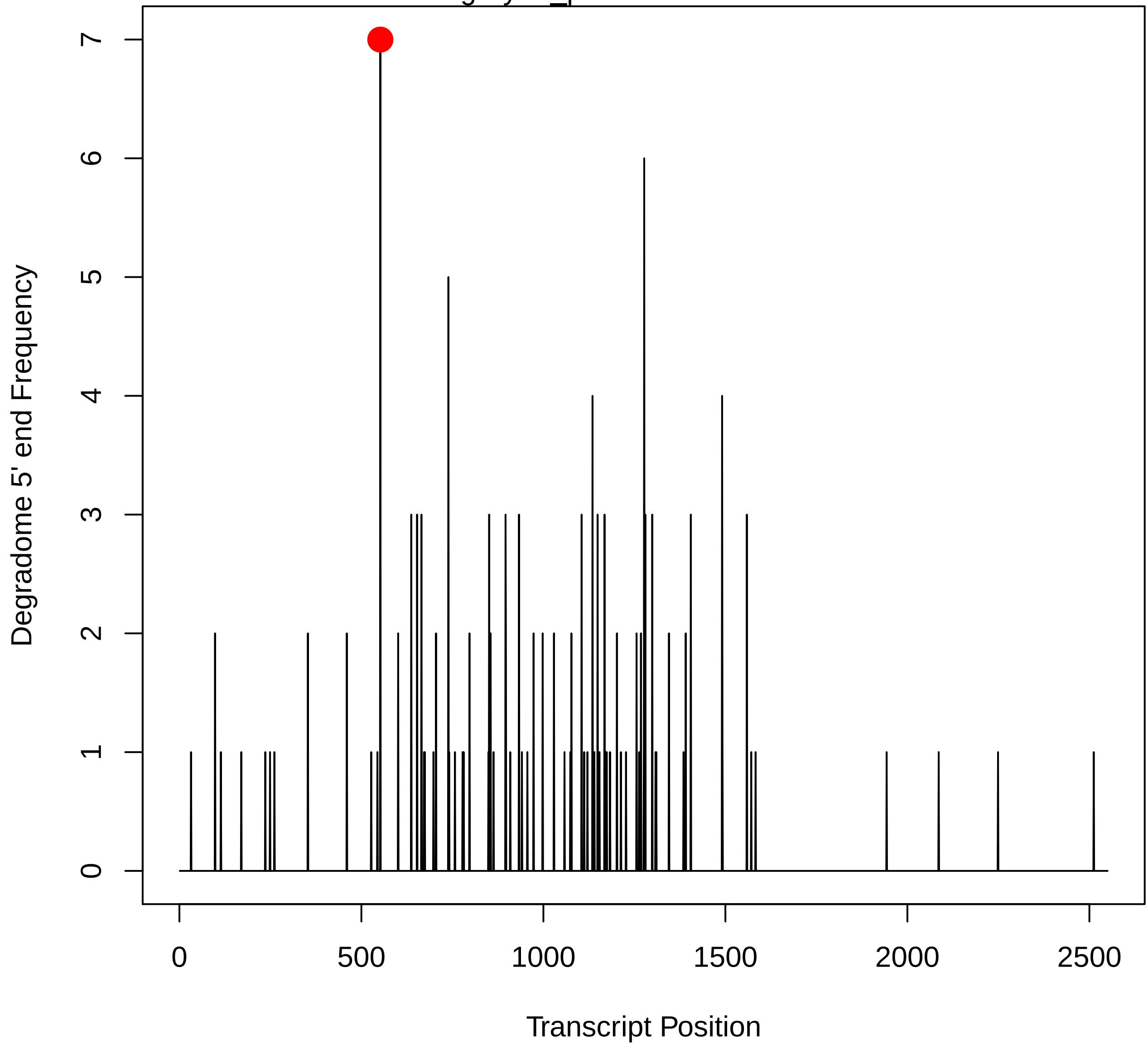

Supplement: Supplementary file 4 [file Data_Sheet_4.zip › Sit-miR171g_Seita.3G274300.1_552_TPlot.pdf]
